# Book Reviews

**Published:** 1905-04

**Authors:** 


### BOOK REVIEWS.

---

LEA'S SERIES OF MEDICAL EPITOMES. Edited by Victor C. Pedersen, M.D.

HOLLIS' EPITOME OF MEDICAL DIAGNOSIS. A Manual for Students and Physicians. By Austin W. Hollis, M.D., Attending Physician to St. Luke's Hospital; to the New York Dispensary, etc. In one 12mo volume of 319 pages, with 13 illustrations. Cloth, $1.00 net. Lea Brothers & Co., Publishers, Philadelphia and New York, 1905.

As each volume of *Lea's Series of Medical Epitomes* appears, the conviction is strengthened that authors, editor and publishers are using their combined efforts to place before the medical world a set of compendious manuals to which the word "best" exactly applies. For the student preparing for examinations, the candidate who is brushing up for appearance before his State Board, or for the physician who wishes a handy volume to slip into his pocket or under the cushion of his carriage seat, so that at odd moments he may refresh his memory on forgotten details or post himself on the most recent knowledge on any medical subject, the volumes of this series have proved to be far in advance of any previous attempt. Dr. Hollis' volume on Medical Diagnosis, just published, is the fifteenth of the series. It naturally does not claim originality, but it embodies an earnest effort to give a clear, accurate compendious covering of the essentails of its subject, presented with a due sense of the relative importance of its various branches.

Diseases and abnormal conditions are taken up in regular sequence, and physical and clinical signs and symptoms are clearly pointed out with full explanations of their significance.

In addition to physical methods, the author gives directions for laboratory investigations, blood tests, bacteriological and chemical examinations, etc., and as one goes carefully through the book the wonder grows at the enormous amount of clear-cut, modern, well-arranged information which has been compressed between its covers.

Illustrations are used throughout the volume wherever the understanding can be better helped by the combination of text and pictures, and the price of the volume ($1.00), based upon the certainty of a very wide usage, is low enough for every student's purse.

